# Platycodin D, a bioactive component of *Platycodon grandiflorum*, induces cancer cell death associated with extreme vacuolation

**DOI:** 10.1080/19768354.2019.1588163

**Published:** 2019-03-05

**Authors:** Daun Jeon, Seung-Woo Kim, Hong Seok Kim

**Affiliations:** a Department of Molecular Medicine, Inha University College of Medicine, Incheon, Republic of Korea; b Department of Biomedical Sciences, Inha University College of Medicine, Incheon, Republic of Korea

**Keywords:** Cancer, cell death, Platycodin D, vacuolation

## Abstract

Platycodin D (PD) is a major active component of the roots of *Platycodon grandiflorum (Jacq.) A.DC.* and possesses multiple biological and pharmacological properties, including anti-cancer activity. The aim of this study was to characterize PD-induced cytoplasmic vacuolation in human cancer cells and investigate the underlying mechanisms. PD-induced cancer cell death was associated with cytoplasmic pinocytic and autophagic vacuolation. Cellular energy levels were decreased by this compound, leading to the activation of AMP-activated protein kinase (AMPK). Additionally, compound C, an inhibitor of AMPK, completely prevented PD-induced vacuolation. These results suggest that PD induces cancer cell death, associated with excessive vacuolation through AMPK activation when cellular energy levels are low. Therefore, our findings provide a mechanistic rationale for a novel combinatorial approach using PD to treat cancer.

## Introduction

Cancer poses a significant worldwide health problem in both economically developing and developed countries despite advances in its treatment (Torre et al. 2015). Therefore, new anticancer agents with better efficacy and fewer side effects are required. A large number of plant-derived natural chemicals possess anticancer properties and have been implicated in cancer prevention and treatment (Nobili et al. 2009).

Platycodin D (PD) is one of the main saponins extracted from the root of *Platycodon grandiflorum (Jacq.) A.DC*., which has been used for decades as a traditional prescription to eliminate phlegm, relieve cough, reduce inflammation, lower blood pressure and blood sugar levels, and for weight loss; it has also been used to treat tumors and improve human immunity (Nyakudya et al. 2014; Zhang et al. 2015). PD possesses immunostimulatory (Xie et al. 2008), anti-inflammatory (Ahn et al. 2005), anti-obesity (Lee et al. 2010), and anti-atherogenic (Wu et al. 2012) activities. Particularly, PD exerts potent anti-cancer activity against many types of cancers (Khan et al. 2016).

**Figure 1.**
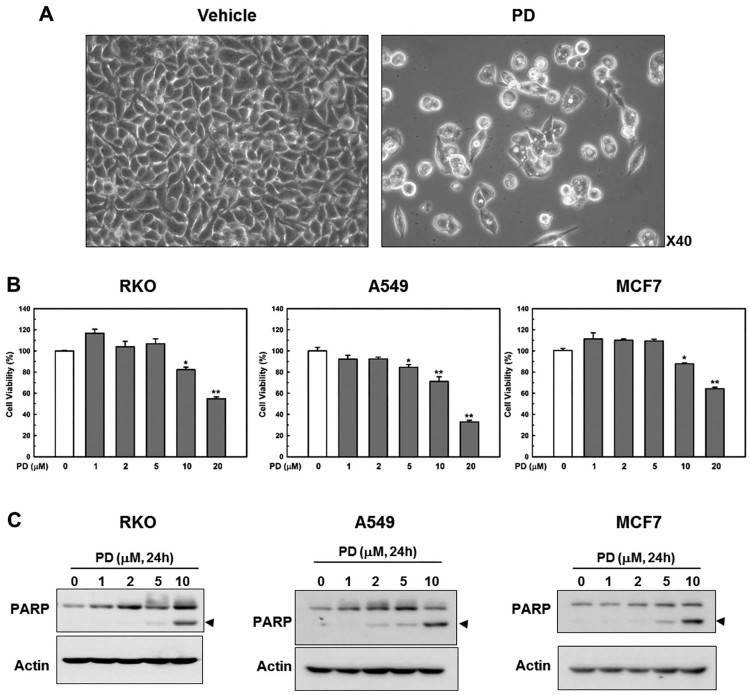
**PD induces cytoplasmic vacuole formation and death in human cancer cells.** (**A**) RKO colorectal cancer cells were treated with vehicle or PD (10 µM) and then examined by phase-contrast microscopy after 48 h. (**B + C**) RKO colorectal cancer cells, A549 lung adenocarcinoma epithelial cells and MCF7 breast adenocarcinoma cells were treated with different concentrations of PD for 24 h. Cell death was assessed with a CellTilter 96 Aqueous One Solution Cell Proliferation Assay kit and by PARP cleavage. Cell viability was significantly different from the control: *p < 0.05; **p < 0.01.

In a preliminary study, we observed the development of profuse, lucent cytoplasmic vacuoles that were readily detected by phase-contrast microscopy (Figure 1(a)) in PD-treated cells, followed by cell death (Figure 1(b + 1c). PD has been suggested to induce autophagy (Li et al. 2015; Zhao et al. 2015); consequently, PD-induced cytoplasmic vacuoles are considered autophagic (Li et al. 2015). However, detailed characterization of these cytoplasmic vacuoles and the mechanisms underlying their development remain unclear.

In this study, we found that PD-induced cancer cell death, associated with the development of pinocytic and autophagic vacuoles, occurs because of AMP-activated protein kinase (AMPK) activation. Our results reveal the mechanisms underlying PD-induced cell death and highlight the potential for developing PD as an anti-cancer agent.

## Materials and methods

### Materials

Platycodin D was obtained from Sigma-Aldrich (St. Louis, MO, USA). Anti-PARP, anti-LC3B, anti-beclin1, anti-phospho-AMPKα (Thr172), and anti-AMPKα antibodies were obtained from Cell Signaling Technology (Danvers, MA, USA), and the anti-β-actin antibody was obtained from Santa Cruz Biotechnology (Dallas, TX, USA).

#### Cell culture

RKO human colorectal cancer cells, A549 human lung adenocarcinoma epithelial cells, and MCF7 human breast adenocarcinoma cells were kindly provided by Dr. Heon Joo Park (Department of Microbiology, Inha University College of Medicine, Incheon, Republic of Korea) and cultured in Dulbecco’s modified Eagle’s medium (HyClone Laboratories, Logan, UT, USA) supplemented with 10% fetal bovine serum (HyClone Laboratories) and 1% antibiotics (Thermo Fisher Scientific, Waltham, MA, USA). Cells were incubated at 37°C in a humidified atmosphere containing 5% CO_2_.

#### Cell viability assay

Cell viability was assessed using the Cell Titer 96® AQueous One Solution cell proliferation assay kit (Promega Corporation, Madison, WI, USA) according to the manufacturer’s instructions. Absorbance was measured at 490 nm with a Multiskan^™^ GO microplate spectrophotometer (Thermo Fisher Scientific).

#### Western blot analysis

Cells were washed with ice-cold phosphate-buffered saline and lysed on ice in RIPA lysis buffer [50 mM Tris-HCl (pH 7.5), 150 mM NaCl, 1% Nonidet P-40, 0.1% sodium dodecyl sulfate, and 0.5% sodium deoxycholate] supplemented with protease and phosphatase inhibitors. Aliquots containing equal amounts of protein were loaded and separated by SDS-PAGE. The proteins were then transferred to nitrocellulose membranes (Bio-Rad, Hercules, CA, USA) and probed using the indicated antibodies. Protein bands were detected by chemiluminescence on a ChemiDoc gel imaging system (Bio-Rad).

#### Small-interfering RNA (siRNA) transfection

Synthetic beclin-1-specific siRNA, siBeclin1 (5′-GAGAUCUUAGAGCAAAUGA-3′), was purchased from Bioneer (Daejeon, Republic of Korea). Non-specific siRNA (Bioneer) was used as a negative control. RKO cells were seeded in 60-mm dishes, grown to ∼80% confluence, and transfected with the siRNA duplexes using Lipofectamine® RNAiMAX (Thermo Fisher Scientific) according to the manufacturer’s recommendations. To determine the extent of siRNA inhibition, expression of beclin-1 in the transfected cells was assessed by western blotting.

#### Uptake of fluorescence-labeled dextrans

The dextran-Alexa Fluor 594 (10,000 MW) tracer was purchased from Thermo Fisher Scientific. To evaluate the cellular uptake of the tracer, the cells were washed twice with phenol red-free DMEM containing 10% fetal bovine serum and then incubated with the tracer (0.5 mg/mL) in the same medium for 8 h (Overmeyer et al. 2008). The cells were washed twice with the same medium without the tracer, and then images of live cells were acquired by laser-scanning confocal microscopy (TE2000-E, Nikon, Tokyo, Japan).

#### Adp/ATP ratio assay

The ADP/ATP ratio was measured based on a luciferin-luciferase reaction using an ADP/ATP ratio assay kit (Sigma-Aldrich) following the manufacturer’s instructions. Luminescence was read on a VICTOR X Light luminescence plate reader (PerkinElmer, Waltham, MA, USA).

#### Electron microscopy

Thin sections of cells embedded in EMbed 812 (Electron Microscopy Sciences, Hatfield, PA, USA) were counterstained with uranyl acetate and lead citrate, and then examined under a Hitachi H-7100 transmission electron microscope (Tokyo, Japan).

#### Statistics

Data were analyzed using analysis of variance (Sigma Stat 12.0, Systat Software, San Jose, CA, USA) with parametric or nonparametric *post hoc* analysis, and multiple comparisons were made by using the least significant difference method. All data are presented as the mean ± SE of at least three independent experiments. The results were considered statistically significant if *p < *0.05.

## Results

### Pd induces development of both autophagic and pinocytic vacuoles

PD-induced cytoplasmic vacuoles are considered autophagic (Li et al. 2015). Further, we observed an increase in LC3 conversion (from LC3-I to LC3-II) (Figure 2(a)). Therefore, we examined whether PD-induced cytoplasmic vacuolation occurred solely because of autophagy. We knocked-down beclin1, one of the first mammalian autophagy effectors (Sinha and Levine 2008), with a specific siRNA in RKO cells (Figure 2(b)) and assessed PD-induced vacuolation. As shown in Figure 2(c), beclin1 knockdown failed to prevent cytoplasmic vacuolation, indicating that most vacuoles were not autophagic.Figure 2.**PD induces development of both autophagic and pinocytic vacuoles**. (**A**) RKO cells, A549 cells, and MCF7 cells were treated with different concentrations of PD for 24 h, and LC3B protein levels were analyzed by western blotting. (**B**) RKO cells were transfected with nonspecific siRNA (siControl) or beclin1-specific siRNA (siBeclin1), and the protein expression of beclin1 was analyzed by western blotting. (**C**) Percentage of vacuolated cells was determined by counting 500 cells in random photomicrographs of the control and beclin1-knockdown cultures. Results shown are the mean ± SE of four independent experiments. (**D**) Cells were treated with PD (5 µM) to induce vacuolation and observed by electron microscopy after 24 h. Some vacuoles contained unidentified inclusions or small quantities of amorphous electron-dense (A) or electron-lucent (*) material. Nu, nucleus. Bar, 500 nm. (**E**) Cells preincubated with PD (5 µM) for 16 h were incubated for 8 h with fluid-phase tracer dextran-Alexa Fluor 594 (red). Merged images of the phase-contrast and fluorescence micrographs are also presented. Bar, 20 µm. The data shown are representative of 3 experiments.

**Figure:**
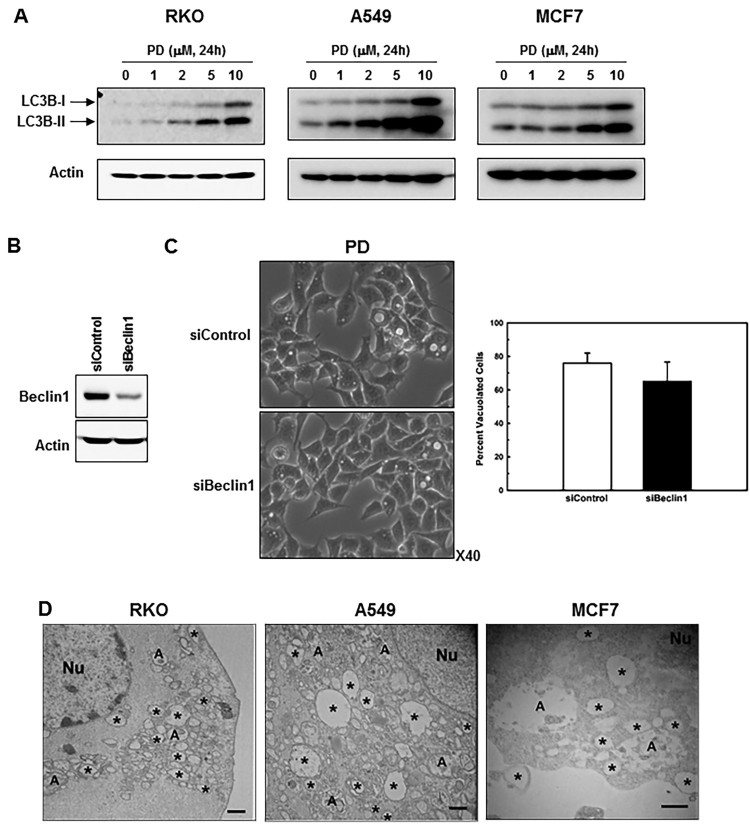

**Figure:**
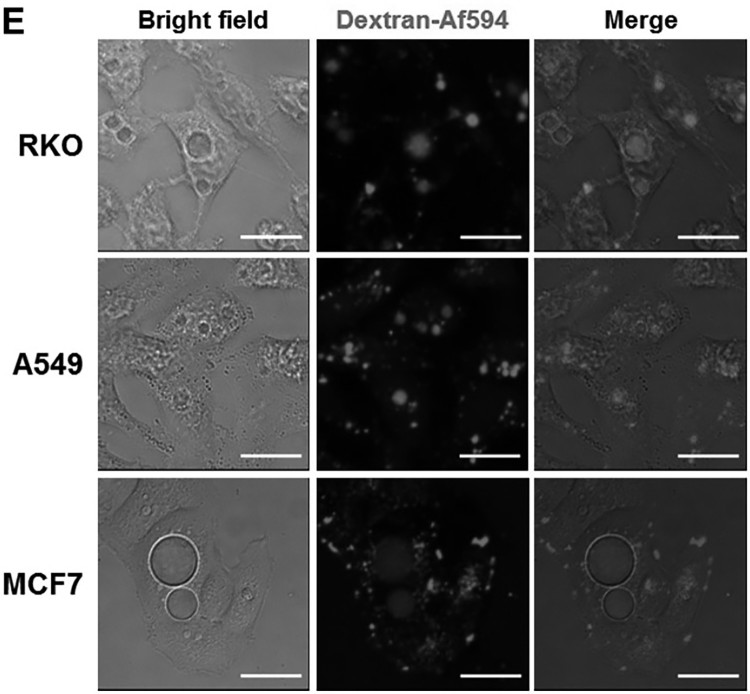

Moreover, electron microscopy of PD-treated cells revealed numerous electron-lucent vacuoles (Figure 2(d)) that were generally devoid of cytoplasmic components or organelles. However, unidentified membranous inclusions or small quantities of amorphous electron-dense material were observed in some cells (Figure 2(d)). The electron-lucent vacuoles were clearly distinct from the description of ‘classic’ autophagosomes described previously (Mizushima et al. 2010).

Several molecules induce cell death associated with the development of macropinocytic vacuoles in cancer cells (Overmeyer et al. 2008; Sun et al. 2017; Lertsuwan et al. 2018). These macropinosomes typically appear as phase-lucent vesicles. To further confirm that the PD-induced vacuoles originated from pinosomes, we investigated fluid-phase endocytosis using Alexa Fluor 594-labeled dextran. As shown in Figure 2(e), PD-induced vacuoles were labeled with fluorescent dextran. These findings, coupled with the morphologic evidence in Figure 2(D), support the identification of PD-induced vacuoles as pinosomes mixed with autophagosomes.

### Pd decreases cellular energy levels, leading to AMPK activation

To explore the molecular pathways underlying the cytoplasmic vacuolation induced by PD, we tested an Erk1/2 inhibitor (PD98059), p38 MAPK inhibitor (SB203580), and JNK inhibitor (SP600125), as Zhao et al. reported that PD induces autophagy through JNK and p38 MAPK activation (Zhao et al. 2015). However, we observed no significant decrease in PD-induced vacuoles (data not shown).

AMPK plays critical roles in autophagy (Mihaylova and Shaw 2011) and pinocytosis (Guest et al. 2008). Moreover, it has been reported that PD containing butanol fraction of *P. grandiflorum* enhances autophagic cell death via AMPK activation, whereas co-treatment with Compound C, an inhibitor of AMPK, decreases LC3-II level (Yim et al. 2016).

**Figure 3.**
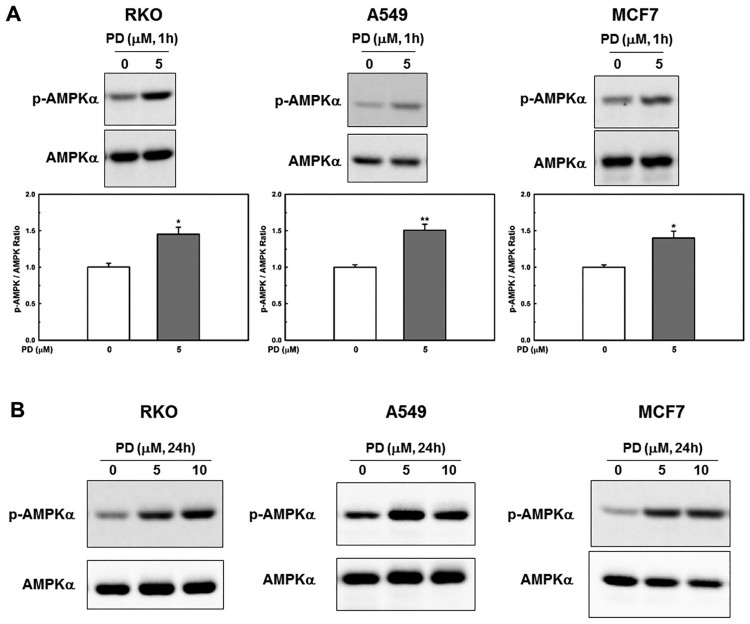
**PD decreases the cellular energy level.** (**A**) Cells were treated with PD (5 µM) for 1 h, after which the activation of AMPKα was evaluated by western blot analysis. Results shown are the mean ± SE of 3 independent experiments. *p < 0.05, **p < 0.01 *vs* control. (**B**) Cells were treated with different concentration of PD for 24 h. Activation of AMPKα was assessed by western blotting.

Therefore, we examined whether PD activates AMPK in cancer cells and observed that PD significantly increased the level of active AMPK (Figure 3(a)) at 1 h and maintained this level for 24 h (Figure 3(b)).

AMPK, a well-known sensor of cellular energy status (Hardie 2011), is activated when intracellular ATP levels decrease (Mihaylova and Shaw 2011). Therefore, we examined whether PD decreases cellular energy levels by measuring the ADP/ATP ratio. An increase in the ADP/ATP ratio, which indicates a decrease in cellular energy status, displaces the adenylate kinase reaction towards ATP and AMP production (Hardie et al. 2012). As expected, PD treatment significantly increased the ADP/ATP ratio (Figure 4(a)). In addition, low ATP levels were maintained for 24 h in PD-treated cells (Figure 4(b)), indicating that PD decreased cellular energy levels.Figure 4.**PD induces sustained activation of AMPKα and PD-induced vacuoles are AMPK-dependent.** (**A**) Cells were treated with PD (5 µM) for 1 h, ADP/ATP ratios were determined using an ADP/ATP ratio assay kit. (**B**) Cells were treated with vehicle or PD (5 µM) for 24 h. Cellular ATP levels were measured. Results shown are the mean ± SE of 3 independent experiments. **p < 0.01 *vs* vehicle control. (**C**) Cells were pre-treated with or without 10 µM compound C (CC) for 1 h before exposure to 5 µM PD for an additional 24 h. Percentage of vacuolated cells was determined by counting 500 cells in random photomicrographs. Results shown are the mean ± SE of 4 independent experiments. **p < 0.01 *vs* PD only. (**D**) Cells were pre-treated with or without 10 µM compound C (CC) for 1 h before exposure to 5 µM PD for an additional 48 h. Cell death was assessed with a CellTilter 96 Aqueous One Solution Cell Proliferation Assay kit. *p < 0.05; **p < 0.01.

**Figure:**
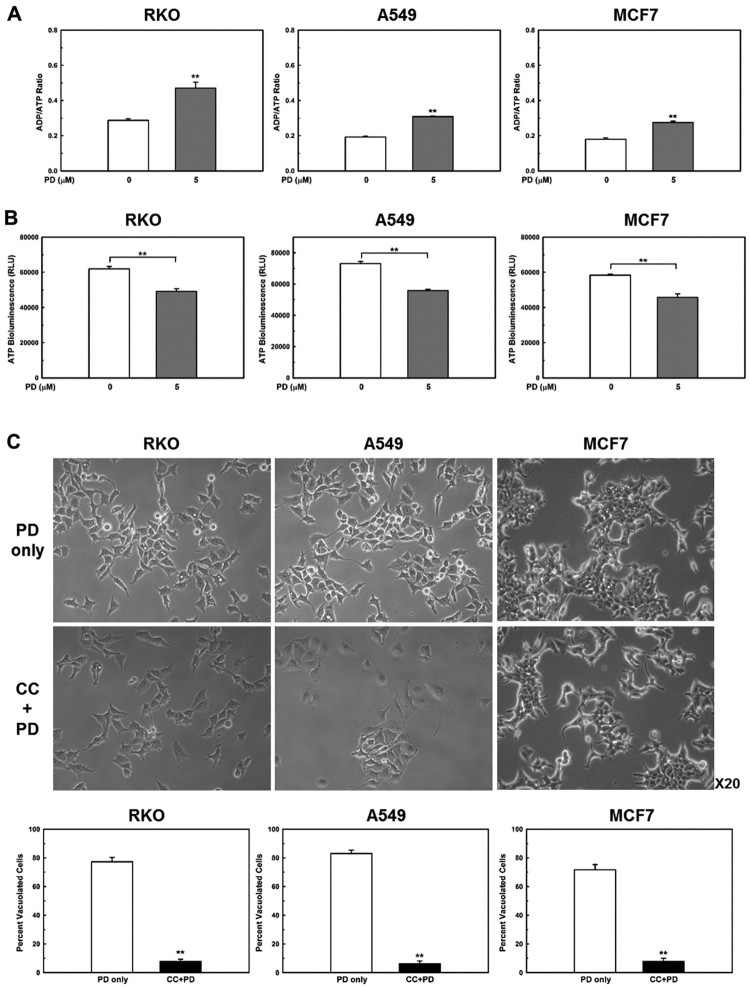

**Figure:**
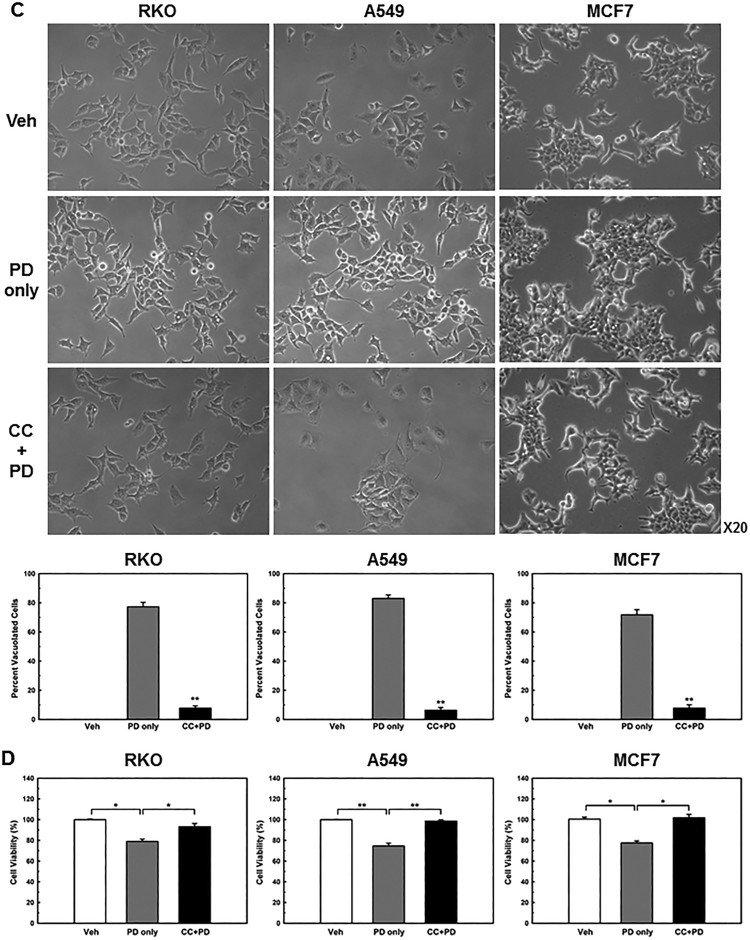

**Figure 5.**
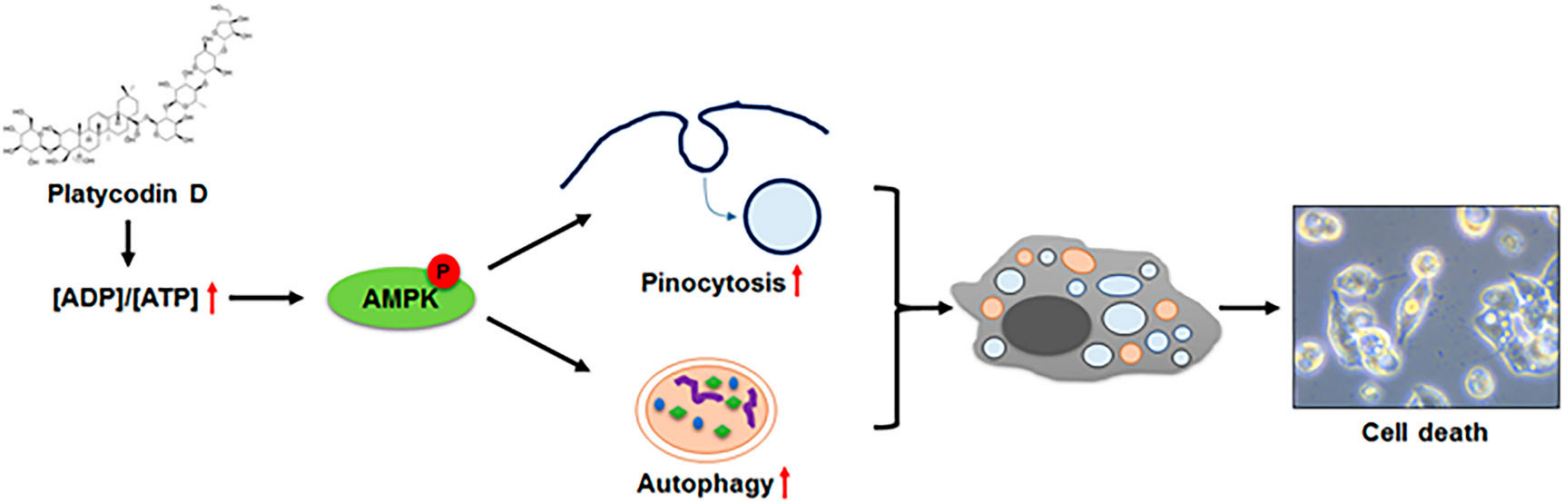
**Schematic model of the molecular mechanisms associated with PD-induced vacuolation and cell death in cancer cells.** According to this model, PD decreased cellular energy levels, which activated the AMPK signaling pathway. Activation of the AMPK signaling pathway contributed to the accumulation of pinosomes and autophagosomes, which are possible upstream signaling pathways of vacuolated cell death induced by PD. The schematic model agrees with the results and conclusions of this study.

To further confirm that PD-induced vacuolation was mediated by AMPK activation, we pre-treated the cancer cells with compound C (10 µM for 1 h), a well-known AMPK inhibitor, before treatment with PD (5 µM for 24 h); compound C completely prevented PD-induced vacuolation (Figure 4(c)). In addition, inhibition of AMPK activation markedly suppressed PD-induced cell death (Figure 4(d)). These findings strongly suggest that PD decreases cellular energy levels, which activates AMPK and results in the accumulation of pinocytic and autophagic vacuoles (Figure 5).

## Discussion

Although chemotherapy is one of the most common treatments for cancer, its effectiveness is limited by drug resistance (Holohan et al. 2013) that results from the high adaptability of cancer cells (Debatin and Krammer 2004). Therefore, combination therapy to block multiple pathways is a cornerstone of cancer therapy (Yap et al. 2013). Although a combinatorial approach with natural compounds is a promising approach for preventing drug resistance (by affecting more than one target) and enhancing the potency of chemotherapy (through chemosensitization), it is important to define the mechanisms of action of these natural compounds.

PD is a potential anti-cancer compound that has been shown to exhibit broad-spectrum cytotoxicity against a wide range of cancer cell lines (Khan et al. 2016). However, further studies are required to establish its mechanism of action for effective combination therapy.

Autophagy is an evolutionarily highly conserved catabolic process that plays a vital role in the degradation of misfolded proteins and damaged organelles (Yang and Klionsky 2010; Sui et al. 2014). It plays a very important role in various physiological and pathological conditions such as cancer (Yang et al. 2011; Panda et al. 2015). Numerous studies have shown that autophagic cell death or type II programed cell death is an alternative mechanism of cancer cell death in apoptosis-resistant cells. The effect of PD-induced autophagy is controversial. Li et al. reported that PD induces protective autophagy in HepG2 hepatocellular carcinoma cells (Li et al. 2015), whereas Yim et al. suggested that PD enhances autophagic cell death in human lung cancer cells (Yim et al. 2016). The present study showed that PD promotes cancer cell death by accumulating not only autophagic but also pinocytic vacuoles. The characteristic feature of autophagic cell death is the proliferation of autophagosomes and autolysosomes that engulf cytoplasm and organelles and cannibalize the cell (Gozuacik and Kimchi 2004; Lockshin and Zakeri 2004). In PD treated cells, the large pinocytic vacuoles that eventually fill the degenerating cells are morphologically distinct from autophagosomes. Specifically, the vacuoles were phase and electron lucent, and were bound by a single membrane (Figure 2(d)), rather than the typical double membrane of autophagosomes. Although autophagosomes seem to accumulate in parallel with the pinocytic vacuoles, our studies with beclin-1 knockdown cells suggest that excessive vacuolation and cell death induced by PD can occur independent of the autophagy machinery. Thus, in this case, autophagy may reflect the attempt of the cells to survive under the adverse metabolic conditions created by rampant pinocytic vacuole accumulation rather than being a direct cause of cell death (Mathew et al. 2007).

Methuosis is one of the most recent additions to the list of nonapoptotic cell death phenotypes. The name, which is derived from the Greek methuo (to drink to intoxication), was selected because the most prominent attribute in cells undergoing this form of death is the accumulation of large fluid-filled cytoplasmic vacuoles that originate from macropinosomes (Overmeyer et al. 2008; Overmeyer et al. 2011, Sun et al. 2017). Although methuosis is distinct from autophagy and other non-apoptotic forms of death, the amount of LC3-II increases on Western blots (Overmeyer et al. 2008). This could reflect either an increase in autophagosome biogenesis (stimulation of cellular macroautophagy pathways) or a decreased lysosomal turnover of LC3-II. Taken together, our results suggest that PD-induced cell death with excessive vacuoles is methuosis.

Rab7 GTPase has been suggested as a common modulator in endocytosis and autophagy (Hyttinen et al. 2013) because Rab7 designates the maturation of endosomes and autophagosomes (Maday et al. 2012, Hyttinen et al. 2013) and participates in the fusion step with lysosomes (Agola et al. 2012). We therefore examined Rab7 levels in PD-treated cancer cells, but no changes were detected (data not shown).

The AMPK signaling cascade has gained attention *in vitro* and *in vivo* anti-cancer studies (Kim & He 2013, Zadra et al. 2015). Consistent with this, PD activated AMPK, which mainly regulated vacuolation and cell death (Figures 3 and 4c+d) even though decrease of ATP levels was only about 20–25% by PD treatment (5μM, 24 h) (Figure 4(b)). Suggesting that sustained activation of AMPK by loss of ATP balance might be more important than absolute ATP decrease in PD-induced cell death.

Interestingly, the chemotherapeutic agent sunitinib has been shown to inhibit AMPK (Laderoute et al. 2010), suggesting that combinatorial treatment of sunitinib and PD would be ineffective. Therefore, this study provides a rationale for combining PD with conventional anticancer agents to target AMPK for improving chemotherapy in various cancers.
